# Blackberry and Blueberry Anthocyanin Supplementation Counteract High-Fat-Diet-Induced Obesity by Alleviating Oxidative Stress and Inflammation and Accelerating Energy Expenditure

**DOI:** 10.1155/2018/4051232

**Published:** 2018-07-02

**Authors:** Tao Wu, Yufang Gao, Xueqi Guo, Min Zhang, Lingxiao Gong

**Affiliations:** ^1^Key Laboratory of Food Nutrition and Safety (Tianjin University of Science and Technology), Ministry of Education, Tianjin 300457, China; ^2^Tianjin Key Laboratory of Food Nutrition and Safety, Tianjin University of Science and Technology, Tianjin 300457, China; ^3^Beijing Advanced Innovation Center for Food Nutrition and Human Health, Beijing Technology & Business University, Beijing, China

## Abstract

Many studies indicate that an anthocyanin-rich diet has beneficial effects preventing metabolic disease. In the present study, the molecular mechanism underlying the antiobesity effect of consuming blackberry anthocyanins (BLA) and blueberry anthocyanins (BBA) was investigated in high-fat-diet- (HFD-) fed C57BL/6 mice. Sixty mice were administered a low-fat diet (LFD), a HFD, or a HFD plus orlistat, and BLA or BBA in their daily food for 12 weeks. As a result, the consumption of BLA and BBA inhibited body weight gain by 40.5% and 55.4%, respectively, in HFD-fed mice. The BLA and BBA treatments markedly reduced serum and hepatic lipid levels and significantly increased hepatic superoxide dismutase and glutathione peroxidase activities. In addition, the treatments effectively increased fecal acetate and butyrate levels and significantly attenuated expression of tumor necrosis factor TNF-*α*, interleukin-6, and nuclear factor-kappaB genes. Moreover, gas chromatography time-of-flight mass spectroscopy results suggested that BLA and BBA significantly affected the hepatic lipid and glucose metabolic pathways, including glycerophospholipid metabolism, glutathione metabolism, and the insulin-signaling pathway. Therefore, BLA and BBA ameliorated diet-induced obesity by alleviating oxidative stress and inflammation and accelerating energy expenditure.

## 1. Introduction

Obesity has been officially recognized as a chronic disease and continues to be a major global health challenge. Obesity results from an extreme imbalance between energy expenditure and energy consumption [1, 2]. Phentermine, orlistat, liraglutide, lorcaserin, phentermine/topiramate extended release, and naltrexone sustained release (SR)/bupropion SR have been approved as antiobesity medications [3, 4]. However, these drugs cause unwanted side effects [3, 5]. Therefore, food components to counteract obesity are needed.

Anthocyanins are water-soluble polyphenolic compounds commonly found in the daily diet, particularly in pigmented fruits and vegetables [6, 7]. These substances have attracted attention recently for their potential pharmacological activities, including anti-inflammatory, antioxidative, and antiobesity effects [8, 9]. Several studies have demonstrated that anthocyanin-rich blueberry [10, 11], mulberry [12, 13], wolfberry [14], cherry [15, 16], red cabbage microgreen [17], black rice [18, 19], purple corn [20], and sweet potato [21, 22] have beneficial effects on body weight management in various models. However, the specific molecular mechanisms whereby anthocyanins alter bodyweight gain are not fully understood. Possible mechanisms include attenuation of high-sensitivity C-reactive protein, reduced expression of sodium-dependent glucose transporter 1, lowered food availability, and normalization of the ratio between beneficial and pathogenic bacteria [23–26].

Blackberry (*Rubus* sp.) and blueberry (*Vaccinium ashei*) are desired fruits because of their aromatic taste and health benefits [10, 27]. Previous studies have suggested that blackberry and blueberry anthocyanins might prevent obesity [8, 27]. However, the specific molecular mechanism underlying the antiobesity effect remains elusive. Therefore, this study investigated the potential mechanisms of blackberry anthocyanin (BLA) and blueberry anthocyanin (BBA) in diet-induced obese C57BL/6 mice.

## 2. Materials and Methods

### 2.1. Preparation of the Purified Anthocyanins

BLA and BBA were obtained from our laboratory. BLA consisted of cyanidin-3-glucoside (93.32%) and peonidin-3-glucoside (6.68%), while BBA was composed of cyanidin-3-glucoside (51.24%), cyanidin-3-rutinoside (42.31%), and peonidin-3-glucoside (6.91%).

### 2.2. Animals and Diets

All experimental animal procedures were approved by the Animal Care and Use Committee of Tianjin University of Science and Technology (20161012). Sixty male C57BL/6 mice (24 days of age) were obtained from the Beijing Laboratory Animal Center. The mice were allowed to acclimate for 7 days and were subsequently maintained on a 12 h light/dark cycle with free access to water and a diet. All experimental animals were divided randomly into five groups (*n* = 12): the LFD group—mice fed a low-fat diet (10% of calories from fat); the HFD group—mice fed a high-fat diet (45% of calories from fat); the OC group—mice fed the HFD plus orlistat at a dose of 40 mg/kg food; the BLA group—mice fed the HFD plus BLA at a dose of 200 mg/kg food; and the BBA group—mice fed the HFD plus BBA at a dose of 200 mg/kg food. The human equivalent of the anthocyanin doses was ~2 mg/kg body weight. The detailed composition of the animal diets is provided in Supplementary Table 1. All experimental mice were deprived of their diet for 12 h in week 12, anesthetized with ketamine HCl/xylazine, and killed by decapitation. Serum, liver, kidney, and adipose tissue samples were collected immediately and stored at −80°C until use.

### 2.3. Biochemical Analyses of Serum and Liver Samples

Serum total cholesterol (TC), triglycerides (TGs), low-density lipoprotein cholesterol (LDL-C), and high-density lipoprotein cholesterol (HDL-C) levels were analyzed by a Sysmex Analyzer KX-21 according to the manufacturer's instructions. The hepatic lipid profiles were determined according to the method of Folch et al. [28]. Serum and hepatic superoxide dismutase (SOD) activity, malondialdehyde (MDA) level, and glutathione peroxidase activity (GP_X_) level were characterized by the hydroxylamine method, the thiobarbituric acid method, and a cellular GP_X_ assay kit, respectively.

### 2.4. Fecal Short-Chain Fatty Acid (SCFA) Analysis

The composition and concentration of minor SCFAs, including acetic acid, propionic acid, butyric acid, isobutyric acid, isovaleric acid, and valeric acid, were characterized in mouse feces according to the method of Periago et al. [29].

### 2.5. Quantitative Real-Time Polymerase Chain Reaction (PCR)

Liver samples were ground in liquid nitrogen, and total RNA was extracted using TRIzol and purified in an RNeasy column. cDNA was obtained by reverse transcription from total RNA. Gene expression of interleukin-6 (IL-6), tumor necrosis factor-*α* (TNF-*α*), and nuclear factor-*κ*B (NF-*κ*B) was analyzed by PCR using the One-Step SYBR PrimeScript PLUS RT-PCR kit.

### 2.6. Analysis of Liver Metabolites

Liver metabolite analysis was performed using Agilent 7890 gas chromatography time-of-flight mass spectrometry (GC-TOF/MS) system (Agilent Technologies, Palo Alto, CA, USA) with a capillary Agilent DB-5MS column (J&W Scientific, Folsom, CA, USA). A principal component analysis and orthogonal projections to latent structure discriminant analysis was performed with the SIMCA14.1 software package (V14.1, MKS Data Analytics Solutions, Umea, Sweden). Various hepatic metabolites, including glucose and lipid pathway intermediates, were quantified.

### 2.7. Statistical Analysis

Statistical analyses were performed using SPSS software version 19.0 (SPSS Inc., Chicago, IL, USA). The mean ± standard error of each group was calculated. Significant differences among groups were analyzed by the analysis of variance and a post hoc Duncan's multiple range test. A *p* value < 0.05 was considered significant.

## 3. Results

### 3.1. Effects of BLA and BBA on Body Weight Gain

**Figure 1:**
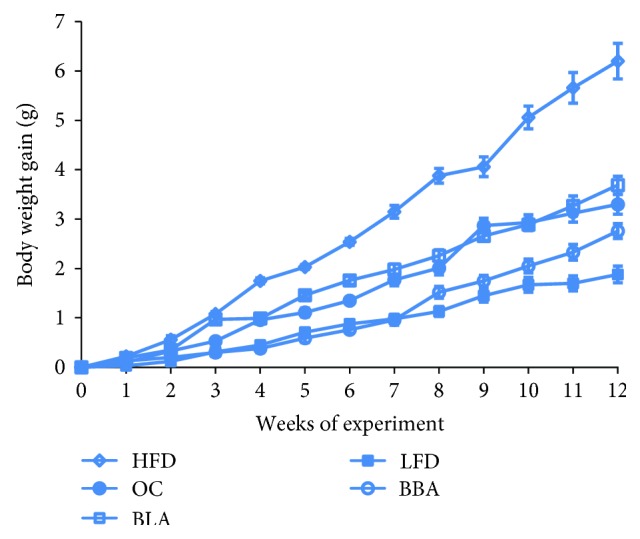
Effects of blackberry anthocyanin (BLA) and blueberry anthocyanin (BBA) consumption on body weight gains. LFD: mice fed with low-fat diet; HFD: mice fed with high-fat diet; OC: mice fed with orlistat; BLA: mice fed with HFD plus BLA at doses of 200 mg/kg food; BBA: mice fed with HFD plus BBA at doses of 200 mg/kg food. Data are presented as mean ± SEM.

C57BL/6 mice were fed a HFD supplemented with BLA or BBA at a dose of 200 mg/kg in daily food for 12 weeks to determine the antiobesity effects of BLA and BBA. At the end of the experiment, BLA, orlistat, or BBA inhibited body weight gain by 40.5%, 53.2%, or 55.4%, respectively, compared with the HFD group (Figure 1). No significant differences were observed in daily food intake (~2.8 g), the liver, or kidney throughout the experiment, whereas BLA and BBA significantly reduced the food utility and effectively increased the weight of liver tissue when expressed as a percentage of body weight (Table 1). Furthermore, the epididymal fat weight of mice in the HFD group increased when compared with mice in the LFD group, and BLA or BBA significantly attenuated this increase in weight.

### 3.2. Effects of BLA and BBA on Serum Lipids and Antioxidants

**Figure 2:**
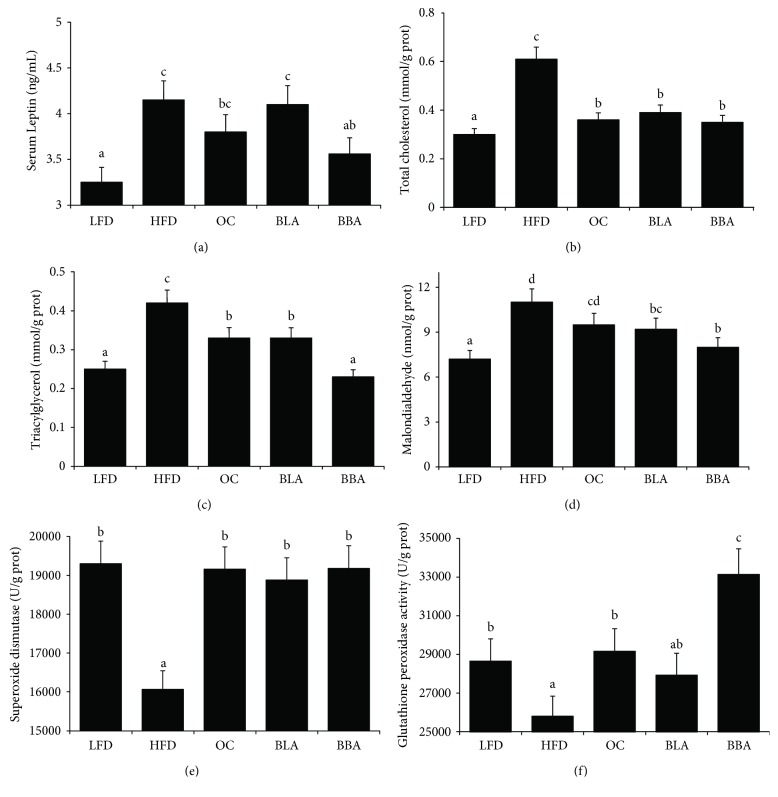
Effects of blackberry anthocyanin (BLA) and blueberry anthocyanin (BBA) consumption on serum leptin and hepatic lipids and antioxidants: (a) leptin; (b) TC; (c) TG; (d) MDA; (e) SOD; (f) GP_X_. LFD: mice fed with low-fat diet; HFD: mice fed with high-fat diet; OC: mice fed with orlistat; BLA: mice fed with HFD plus BLA at doses of 200 mg/kg food; BBA: mice fed with HFD plus BBA at doses of 200 mg/kg food. Data are presented as mean ± SEM and analyzed with ANOVA and post hoc Duncan's multiple range tests (*p* < 0.05). The means marked with lowercase letters are significantly different relative to others.

BLA or BBA supplementation significantly decreased serum TG, TC, LDL-C, and MDA levels and effectively increased GP_X_ activity when compared to the HFD-fed mice (Table 2). Moreover, BLA effectively increased HDL-C levels but did not alter leptin levels or SOD activity. However, BBA decreased for HDL-C, leptin levels, and SOD activity compared to BLA (Table 2 and Figure 2(a)).

### 3.3. Effects of BLA and BBA on Hepatic Lipids and Antioxidants

Hepatic TGs, TC, MDA, SOD, and GP_X_ were characterized at the end of the experiment.  Figure 2 shows that BLA and BBA significantly reduced hepatic lipid and MDA levels and significantly elevated SOD and GPx activities, when compared with mice in the HFD group. Moreover, BBA-treated mice had significantly lower hepatic TG levels and significantly higher GP_X_ activity when compared to BLA-treated mice.

### 3.4. Effects of BLA and BBA on SCFAs

Table 3 shows that all six types of minor SCFAs decreased in the HFD group, compared with the LFD group. BBA and BLA significantly increased the concentrations of acetic acid, propionic acid, butyric acid, and valeric acid but significantly reduced isobutyric acid levels. Furthermore, the levels of acetic acid, propionic acid, and butyric acid increased significantly in BBA-treated mice compared to BLA-treated mice.

### 3.5. Effects of BLA and BBA on Inflammation

**Figure 3:**
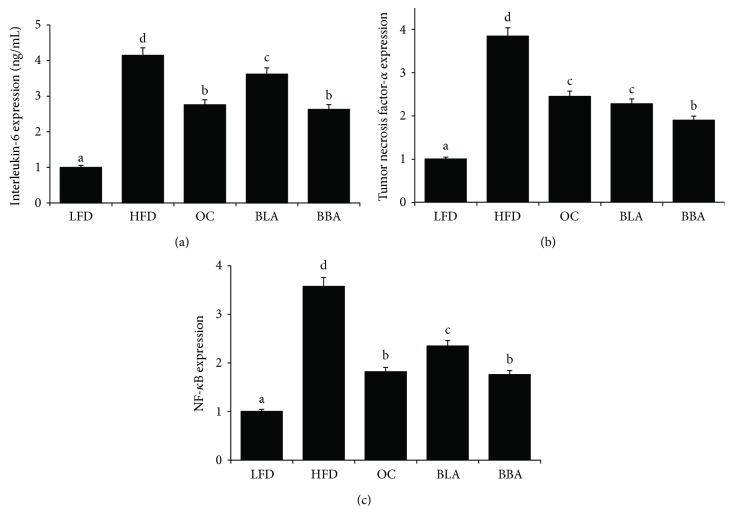
Effects of blackberry anthocyanin (BLA) and blueberry anthocyanin (BBA) consumption on the gene expression of inflammatory cytokine. LFD: mice fed with low-fat diet; HFD: mice fed with high-fat diet; OC: mice fed with orlistat; BLA: mice fed with HFD plus BLA at doses of 200 mg/kg food; BBA: mice fed with HFD plus BBA at doses of 200 mg/kg food. Data are presented as mean ± SEM and analyzed with ANOVA and post hoc Duncan's multiple range tests (*p* < 0.05). The means marked with lowercase letters are significantly different relative to others.

The expression levels of the inflammatory markers such as IL-6, TNF-*α*, and NF-*κ*B in liver tissue were determined by quantitative real-time PCR (Figure 3). Administration of BBA and BLA significantly decreased gene expression levels of IL-6, TNF-*α*, and NF-*κ*B when compared with those observed in HFD-fed mice. Moreover, BBA was more effective in reducing the inflammatory markers than BLA in the current experiment.

### 3.6. Effects of BLA and BBA on Hepatic Metabolites

**Figure 4:**
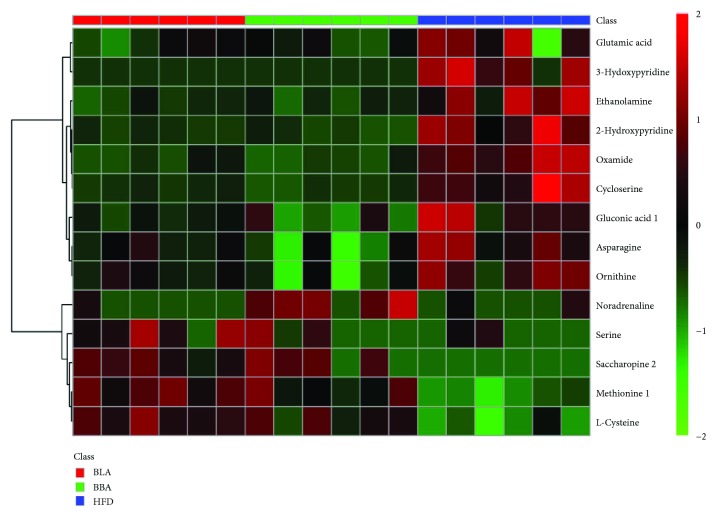
Heat map of the differently expressed metabolites present among BLA/BBA-HFD. HFD: mice fed with high-fat diet; BLA: mice fed with HFD plus BLA at doses of 200 mg/kg food; BBA: mice fed with HFD plus BBA at doses of 200 mg/kg food.

**Figure 5:**
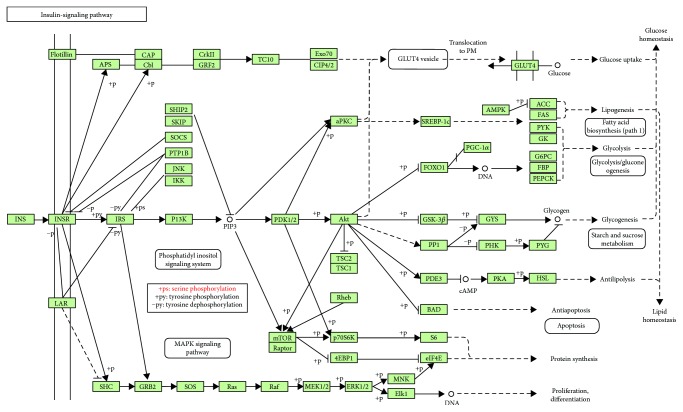
The effect of blackberry anthocyanin (BLA) and blueberry anthocyanin (BBA) consumption on insulin-signaling pathway.

A metabolite analysis of liver tissues was performed by GC-TOF/MS to investigate the hepatic metabolic changes after the BLA and BBA treatments. Sixty-one and sixty-three hepatic metabolites differed significantly in abundance between the BLA, BBA, and HFD groups (Supplementary Tables 2 and 3), respectively. Furthermore, 14 different metabolites were observed among the BLA, BBA, and HFD groups (Figure 4). What is more, 12 common metabolites including nine downregulated metabolites and three upregulated metabolites were found compared with the HFD group. These results indicate that consuming BLA and BBA significantly affected the hepatic lipid and glucose metabolic pathways, including glycerophospholipid metabolism, glutathione metabolism, and the insulin-signaling pathway (Figure 5).

## 4. Discussion

Obesity has become a leading global health problem as it causes a profound impact on morbidity and mortality [1, 30]. Recent findings suggest that fruit- and vegetable-based diets with antioxidant and anti-inflammatory activities, especially anthocyanin-rich diets, are recommended to prevent the development of metabolic diseases [23, 31–33]. In the present study, the molecular mechanism underlying the antiobesity effect of BLA and BBA was investigated in HFD-induced obesity.

R. Prior and R. L. Prior et al. reported that consuming a blueberry extract significantly reduces body weight in diet-induced obese C57BL/6 mice, while the intake of wild blueberry powder or blueberry juice does not alter body weight in obese mice [34, 35]. Wu et al. suggested that artificially planted blueberry, mulberry, and honeysuckle anthocyanins mitigate body weight gain [36–38]. In addition, Johnson et al. reported that an alcohol-free blueberry-blackberry beverage prevents the development of obesity and attenuates fasting blood glucose in C57BL/6J mice [39]. In the current study, consuming purified BLA (mainly cyanidin-3-glucoside) and BBA (mainly cyanidin-3-glucoside and cyanidin-3-rutinoside) at 200 mg/kg in the daily diet effectively inhibited body weight gain, significantly decreased serum and hepatic lipids (TC and TGs), significantly increased antioxidant activities (SOD and GP_X_), and significantly attenuated inflammation. However, BBA accelerated fatty acid decomposition and attenuated liver inflammation more than BLA. These data suggest that structural differences in the anthocyanin sugar moieties may be attributed to the different antiobesity effects [35, 40].

Marques et al. reported that blackberry anthocyanin is quickly absorbed in the stomach in an intact form, while methyl-cyanidin-glucuronide and 3′-methyl-cyanidin-3-glucoside are the main conjugates in plasma and urine samples [41]. Cyanidin-3-glucoside potentiates its conversion into methylated derivatives (Me-Cy-Glucr and 3-Me-Cy3glc) in overweight and obese individuals [42]. It is difficult to determine whether a particular anthocyanin is solely responsible for the antiobesity effect due to the lack of information on bioavailability [43]. Therefore, further studies are needed to identify which compounds reach the target organs.

Accumulating evidence indicates that obesity is associated with systemic oxidative stress and low-grade systemic inflammation, whereas consuming polyphenols attenuates these disease conditions [44, 45]. Our findings from the present study also demonstrate that HFD-fed obese mice exhibited pathophysiological conditions of excessive oxidative stress and inflammation accompanied by obesity. The BLA and BBA supplements significantly decreased serum and hepatic peroxidation (Table 1, Figure 2) and significantly increased hepatic SOD and GPx activities. Furthermore, administering BBA and BLA significantly downregulated IL-6, TNF-*α*, and NF-*κ*B gene expression levels. However, BBA may have improved oxidative stress and inflammation more than BLA (Figures 2 and 3). These results suggest that cyanidin-3-glucoside and cyanidin-3-rutinoside may exhibit a synergistic effect on alleviating oxidative stress and inflammation.

SCFAs, particularly acetate and butyrate, present a balance between fatty acid synthesis, oxidation, and lipolysis [46, 47]. Our data suggest that consuming BBA and BLA did not alter the daily food intake of mouse but markedly decreased the weight of epididymal and retroperitoneal fat and significantly increased the fecal concentrations of acetic acid, propionic acid, and butyric acid. Moreover, the GC-TOF/MS results indicated that BLA and BBA significantly affected the hepatic lipid and glucose metabolic pathways, including glycerophospholipid metabolism, glutathione metabolism, and insulin-signaling pathways (Figure 5). These results may suggest that BLA and BBA prevent high-fat-diet-induced obesity by accelerating energy expenditure [26, 48, 49].

## 5. Conclusion

In conclusion, our data demonstrate that BLA and BBA inhibited body weight gain by 40.5% and 55.4%, respectively. Furthermore, BLA and BBA significantly reduced serum and hepatic lipids; significantly increased hepatic SOD and GP_X_ activities, as well as fecal acetate and butyrate levels; and significantly attenuated the expression of the TNF-*α*, IL-6, and NF-*κ*B genes. Moreover, BLA and BBA significantly affected the hepatic lipid and glucose metabolic pathways, including glycerophospholipid metabolism, glutathione metabolism, and the insulin-signaling pathway. Therefore, BLA and BBA ameliorated diet-induced obesity by alleviating oxidative stress and inflammation and accelerating energy expenditure.

## Figures and Tables

**Table 1:** Effects of BLA and BBA consumption on food utility and tissue weights.

Group	Food utility **(**%**)**	Liver (g/100 g)	Kidney (g/100 g)	Epididymal fat (g/100 g)
LFD	1.36 ± 0.22^ab^	3.69 ± 0.24^b^	1.23 ± 0.07	2.52 ± 0.55^a^
HFD	2.88 ± 0.61^c^	3.34 ± 0.19^a^	1.18 ± 0.08	4.50 ± 1.60^c^
OC	1.55 ± 0.51^b^	3.28 ± 0.06^a^	1.12 ± 0.06	3.35 ± 0.53^b^
BLA	1.67 ± 0.32^b^	3.55 ± 0.14^b^	1.17 ± 0.08	3.59 ± 0.32^b^
BBA	1.02 ± 0.35^a^	3.83 ± 0.10^b^	1.26 ± 0.06	3.34 ± 0.49^b^

Data are presented as mean ± SEM and analyzed with ANOVA and post hoc Duncan's multiple range tests (*p* < 0.05). LFD: mice fed with low-fat diet; HFD: mice fed with high-fat diet; OC: mice fed with orlistat; BLA: mice fed with HFD plus BLA at doses of 200 mg/kg food; BBA: mice fed with HFD plus BBA at doses of 200 mg/kg food. The means marked with superscript letters are significantly different relative to others.

**Table 2:** Effects of BLA and BBA consumption on serum parameter.

Group	TG (mmol/L)	TC (mmol/L)	HDL-C (mmol/L)	LDL-C (mmol/L)	MDA (nmol/mL)	T-SOD (U/mL)	GP_X_ (U/mL)
LFD	1.59 ± 0.26^a^	1.44 ± 0.30^a^	1.95 ± 0.21^ab^	0.55 ± 0.12^a^	4.29 ± 2.21^b^	162.47 ± 16.52^b^	1014.58 ± 64.11^b^
HFD	2.16 ± 0.45^b^	3.05 ± 0.30^d^	1.64 ± 0.30^a^	1.95 ± 0.64^c^	10.00 ± 3.37^a^	133.23 ± 15.38^a^	722.18 ± 43.40^a^
OC	1.67 ± 0.33^a^	2.24 ± 0.32^c^	1.91 ± 0.30^ab^	0.98 ± 0.13^b^	6.53 ± 1.87^b^	153.64 ± 23.69^ab^	1125.73 ± 41.28 ^b^
BLA	1.47 ± 0.28^a^	2.11 ± 0.57^c^	2.12 ± 0.25^b^	1.00 ± 0.36^b^	6.33 ± 3.37^b^	147.30 ± 16.48^ab^	1260.46 ± 72.03 ^c^
BBA	1.50 ± 0.44^a^	1.83 ± 0.16^b^	1.86 ± 0.85^ab^	0.86 ± 0.38^ab^	5.31 ± 0.71^b^	194.74 ± 8.76^c^	1295.18 ± 52.60 ^c^

Data are presented as mean ± SEM and analyzed with ANOVA and post hoc Duncan's multiple range tests (*p* < 0.05). LFD: mice fed with low-fat diet; HFD: mice fed with high-fat diet; OC: mice fed with orlistat; BLA: mice fed with HFD plus BLA at doses of 200 mg/kg food; BBA: mice fed with HFD plus BBA at doses of 200 mg/kg food. The means marked with superscript letters are significantly different relative to others.

**Table 3:** Effects of BLA and BBA consumption on feces SCFA.

Group	Acetic acid	Propionic acid	Butyric acid	Isobutyric acid	Isovaleric acid	Valeric acid
LFD	6.190 ± 0.283^d^	4.416 ± 0.035^c^	4.898 ± 0.750^d^	1.875 ± 0.125^e^	1.223 ± 0.066^c^	1.514 ± 0.107^c^
HFD	0.913 ± 0.189^a^	0.932 ± 0.059^a^	1.691 ± 0.066^a^	0.843 ± 0.052^c^	0.597 ± 0.101^bc^	0.486 ± 0.041^a^
OC	4.221 ± 0.501^c^	6.747 ± 0.463^d^	2.449 ± 0.261^c^	0.977 ± 0.021^d^	0.778 ± 0.142^c^	0.751 ± 0.071^b^
BLA	1.475 ± 0.260^b^	1.411 ± 0.264^b^	2.049 ± 0.032^b^	0.618 ± 0.040^b^	0.471 ± 0.009^a^	0.639 ± 0.064^b^
BBA	5.951 ± 0.507^d^	4.610 ± 0.332^c^	6.672 ± 0.439^e^	0.326 ± 0.027^a^	0.487 ± 0.063^ab^	0.673 ± 0.064^b^

Data are presented as mean ± SEM and analyzed with ANOVA and post hoc Duncan's multiple range tests (*p* < 0.05). LFD: mice fed with low-fat diet; HFD: mice fed with high-fat diet; OC: mice fed with orlistat; BLA: mice fed with HFD plus BLA at doses of 200 mg/kg food; BBA: mice fed with HFD plus BBA at doses of 200 mg/kg food. The means marked with superscript letters are significantly different relative to others.

## Data Availability

The data used to support the findings of this study are available from the corresponding author upon request.

## Supplementary Materials

Supplementary Materials Supplementary Table 1: composition of diets fed to mice. Supplementary Table 2: differentially expressed hepatic metabolites between BLA/HFD. Supplementary Table 3: differentially expressed hepatic metabolites between BBA/HFD.
