# Supplementary material for: Blackberry and Blueberry Anthocyanin Supplementation Counteract High-Fat-Diet-Induced Obesity by Alleviating Oxidative Stress and Inflammation and Accelerating Energy Expenditure
Source: Oxid Med Cell Longev. 2018 Jul 2;2018:4051232. doi: 10.1155/2018/4051232 (PMC6051031; doi:10.1155/2018/4051232)
Supplement: Supplementary Materials — Supplementary Table 1: composition of diets fed to mice. Supplementary Table 2: differentially expressed hepatic metabolites between BLA/HFD. Supplementary Table 3: differentially expressed hepatic metabolites between BBA/HFD. [file 4051232.f1.docx]

**Supplementary Table 1 Composition of diets fed to mice**

**Supplementary Table 2: Differentially expressed hepatic metabolites between BLA/HFD.**

**Supplementary Table 3: Differentially Expressed Hepatic Metabolites between BBA/HFD**

**Supplementary Table 1 Composition of diets fed to mice**

| Ingredient | MD10% Fat | MD45% Fat |
| --- | --- | --- |
| Casein | 18.96 | 23.31 |
| L-cystein | 0.28 | 0.35 |
| Corn starch | 29.86 | 8.48 |
| Maltodextrin | 3.32 | 11.65 |
| Sucrose | 33.17 | 20.14 |
| Cellulose | 4.74 | 5.83 |
| Soybean oil | 2.37 | 2.91 |
| Lard | 1.9 | 20.68 |
| Mineral Mix | 0.95 | 1.16 |
| Phosphate dicalcium | 1.23 | 1.51 |
| Carbonate calcium | 0.5 | 0.64 |
| Potassium citrate, H_2_O | 1.56 | 1.92 |
| Vitamin Mix | 0.95 | 1.16 |
| Choline bitartrate | 0.19 | 0.23 |
| Total | 99.98 | 99.97 |
|  |  |  |
| Energy Composition | 100 | 100 |
| Protein | 20 | 20 |
| Carbohydrate | 70 | 35 |
| Fat | 10 | 45 |

**Supplementary Table 2 Differentially Expressed Hepatic Metabolites between BLA/HFD**

| ID | Peak | Similarity | R.T. | Count | Mass | MEAN HFD | MEAN BLA | VIP | P-VALUE | Q-VALUE | FOLD CHANGE | LOG_FOLDCHANGE |
| --- | --- | --- | --- | --- | --- | --- | --- | --- | --- | --- | --- | --- |
| 137 | uracil | 909 | 11.1678,0 | 36 | 99 | 0.40337607 | 0.22939338 | 1.75310666 | 0.00323482 | 0.04226551 | 1.75844688 | 0.814301755 |
| 102 | Ethanolamine | 864 | 10.1674,0 | 36 | 174 | 0.44009625 | 0.25617922 | 1.86601329 | 0.00693729 | 0.05475712 | 1.71792328 | 0.780665607 |
| 63 | 3-hydroxybutyric acid | 862 | 8.66729,0 | 36 | 147 | 0.76290899 | 0.4007142 | 1.47193954 | 0.01973142 | 0.0899074 | 1.90387309 | 0.928937313 |
| 28 | 2-hydroxypyridine | 853 | 6.94215,0 | 36 | 152 | 0.70276956 | 0.28856816 | 2.09722889 | 0.00185866 | 0.03295543 | 2.43536765 | 1.284139584 |
| 199 | asparagine 4 | 844 | 13.3522,0 | 36 | 115 | 0.36717214 | 0.20614329 | 1.73598195 | 0.00406629 | 0.04631343 | 1.78115006 | 0.832809065 |
| 205 | methionine 1 | 822 | 13.501,0 | 36 | 176 | 0.23154893 | 0.34106633 | 1.90727971 | 0.00211482 | 0.03508784 | 0.67889707 | -0.558735231 |
| 363 | pantothenic acid | 795 | 18.6864,0 | 36 | 201 | 0.02915901 | 0.01824426 | 1.58603461 | 0.01807842 | 0.08616622 | 1.59825628 | 0.676498762 |
| 246 | glutamic acid | 791 | 14.7007,0 | 36 | 246 | 0.5165212 | 0.22588197 | 1.89385399 | 0.000879 | 0.02113843 | 2.28668625 | 1.193258431 |
| 182 | Aminomalonic acid | 787 | 12.8656,0 | 36 | 147 | 0.32811922 | 0.18779557 | 1.75448445 | 0.00673925 | 0.05434474 | 1.74721494 | 0.805057095 |
| 142 | fumaric acid | 782 | 11.3551,0 | 36 | 245 | 0.04208394 | 0.05221646 | 1.40822008 | 0.04141648 | 0.14002624 | 0.8059515 | -0.311235071 |
| 302 | ornithine 1 | 781 | 16.8938,0 | 36 | 142 | 1.5910542 | 1.13097948 | 1.70042585 | 0.00563129 | 0.05166321 | 1.40679316 | 0.492410225 |
| 86 | valine | 778 | 9.46488,0 | 36 | 144 | 1.8404222 | 1.35934853 | 1.54617753 | 0.01453292 | 0.07765337 | 1.35390017 | 0.437121361 |
| 266 | ribose | 771 | 15.3364,0 | 36 | 307 | 0.41654242 | 0.30840545 | 1.71204894 | 0.00748906 | 0.05620245 | 1.35063252 | 0.433635196 |
| 71 | N-Methyl-DL-alanine | 761 | 8.83625,0 | 36 | 130 | 0.12919566 | 0.07076153 | 1.68428206 | 0.00860642 | 0.0590746 | 1.82578948 | 0.868520427 |
| 224 | L-cysteine | 760 | 13.9296,0 | 36 | 220 | 0.08646222 | 0.13996432 | 1.75873141 | 0.00437876 | 0.04757849 | 0.61774472 | -0.694917321 |
| 368 | gluconic acid 1 | 743 | 18.8628,0 | 36 | 292 | 0.01966436 | 0.01282332 | 1.47146613 | 0.02453838 | 0.09912847 | 1.53348374 | 0.616812873 |
| 61 | 3-Hydroxypyridine | 711 | 8.54356,0 | 18 | 152 | 0.06331847 | 2.5761E-08 | 1.96721951 | 0.00676008 | 0.05438896 | 2457885.33 | 21.22898618 |
| 285 | unknown | 571 | 16.2113,0 | 33 | 174 | 0.03812081 | 0.02343477 | 1.30965817 | 0.02338399 | 0.09711024 | 1.62667783 | 0.701928548 |
| 194 | Threitol | 561 | 13.2175,0 | 24 | 217 | 0.00255917 | 2.5761E-08 | 1.99074486 | 0.04871706 | 0.15510657 | 99341.5832 | 16.60011012 |
| 163 | Glutaric Acid | 543 | 12.0748,0 | 36 | 147 | 0.02809806 | 0.00978015 | 1.92740573 | 0.00271996 | 0.03918037 | 2.87296886 | 1.522542356 |
| 309 | unknown | 515 | 17.2394,0 | 20 | 191 | 0.01342986 | 0.00086014 | 1.55252394 | 0.01642826 | 0.08222361 | 15.6135086 | 3.964722869 |
| 143 | unknown | 514 | 11.4377,0 | 36 | 241 | 0.02348732 | 0.05145346 | 2.07911942 | 0.00036126 | 0.01138094 | 0.45647701 | -1.131385897 |
| 179 | 3-Aminoisobutyric acid 1 | 500 | 12.7633,0 | 35 | 174 | 0.03124736 | 0.0409513 | 1.69269394 | 0.01478233 | 0.07828975 | 0.76303703 | -0.390175026 |
| 421 | noradrenaline | 451 | 20.2493,0 | 19 | 174 | 0.00159841 | 0.00712572 | 1.21994082 | 0.01423058 | 0.07686659 | 0.22431575 | -2.156397196 |
| 101 | Dihydroxyacetone | 446 | 9.96849,0 | 21 | 73 | 0.00658844 | 0.04286441 | 1.23735301 | 0.01111906 | 0.06764708 | 0.15370412 | -2.701772288 |
| 237 | beta-Glutamic acid 1 | 442 | 14.4764,0 | 36 | 188 | 0.01968919 | 0.08864883 | 2.19890409 | 0.00420526 | 0.04689079 | 0.22210316 | -2.170698206 |
| 252 | toluenesulfonic acid | 433 | 14.8294,0 | 36 | 229 | 0.10982293 | 0.05973946 | 1.85765274 | 0.00088149 | 0.02117698 | 1.83836492 | 0.878423175 |
| 295 | methionine sulfoxide 2 | 431 | 16.5453,0 | 34 | 128 | 0.10884519 | 0.01831515 | 2.27616482 | 7.1377E-05 | 0.00571589 | 5.94290378 | 2.571168024 |
| 306 | unknown | 417 | 17.1158,0 | 18 | 204 | 0.02131669 | 0.00396052 | 1.33264067 | 0.01019094 | 0.06442832 | 5.38230101 | 2.428223077 |
| 206 | unknown | 399 | 13.5236,0 | 27 | 305 | 0.0038124 | 0.01270507 | 1.6556542 | 0.00902398 | 0.06022011 | 0.300069 | -1.736633826 |
| 493 | Abietic Acid 2 | 392 | 22.2528,0 | 36 | 79 | 0.01831725 | 0.03256845 | 1.41900404 | 0.04790909 | 0.15352471 | 0.56242313 | -0.830272159 |
| 83 | malonic acid 1 | 384 | 9.31636,0 | 35 | 147 | 0.01141606 | 0.00817177 | 1.48618536 | 0.01555821 | 0.08019965 | 1.39701095 | 0.482343327 |
| 160 | DL-Anabasine 1 | 382 | 12.0065,0 | 27 | 239 | 0.01061283 | 0.00093041 | 1.76200417 | 0.00251433 | 0.03791634 | 11.4066109 | 3.511798299 |
| 397 | unknown | 369 | 19.6662,0 | 17 | 173 | 0.00949863 | 2.5761E-08 | 1.61502068 | 0.02562914 | 0.10093697 | 368716.082 | 18.49215082 |
| 151 | 3-Cyanoalanine | 365 | 11.6733,0 | 34 | 141 | 0.01614418 | 0.00965395 | 1.96009845 | 0.00038723 | 0.01162863 | 1.67228647 | 0.741822012 |
| 420 | Octadecanol | 339 | 20.231,0 | 26 | 75 | 0.01172873 | 0.00596492 | 1.06998737 | 0.04966354 | 0.15693387 | 1.96628428 | 0.975471921 |
| 49 | Analyte 53 | 332 | 8.14454,0 | 36 | 245 | 0.02132492 | 0.01354638 | 1.42143609 | 0.02549506 | 0.10071958 | 1.5742157 | 0.654633238 |
| 66 | unknown | 325 | 8.74023,0 | 36 | 128 | 0.22236875 | 0.05044386 | 2.20347125 | 0.00174274 | 0.03189112 | 4.40824214 | 2.14020347 |
| 419 | unknown | 313 | 20.219,0 | 24 | 299 | 2.4558E-08 | 0.00584665 | 1.96258038 | 0.00852164 | 0.05887397 | 4.2003E-06 | -17.86107036 |
| 166 | Biuret 3 | 311 | 12.2171,0 | 30 | 86 | 0.015528 | 0.00194034 | 1.55478413 | 5.3593E-05 | 0.00531296 | 8.00273181 | 3.000492562 |
| 181 | unknown | 300 | 12.861,0 | 26 | 281 | 0.00628311 | 0.00246069 | 1.15707843 | 0.00512726 | 0.0501826 | 2.55339452 | 1.35241646 |
| 133 | oxamide | 299 | 11.0945,0 | 33 | 215 | 0.02894539 | 0.00777974 | 2.24029158 | 9.6946E-06 | 0.00232904 | 3.7206096 | 1.895539018 |
| 227 | unknown | 295 | 14.0406,0 | 23 | 244 | 0.00426798 | 0.00039491 | 2.05062885 | 0.00010956 | 0.00658044 | 10.8075672 | 3.433969896 |
| 123 | unknown | 274 | 10.6905,0 | 36 | 341 | 0.03560597 | 0.02317017 | 1.52254755 | 0.02390913 | 0.09804219 | 1.53671577 | 0.619850347 |
| 529 | Analyte 588 | 274 | 23.8265,0 | 16 | 174 | 0.00650046 | 2.5761E-08 | 1.67927262 | 0.03896296 | 0.13453005 | 252333.559 | 17.94497256 |
| 180 | Citraconic acid 3 | 258 | 12.7741,0 | 18 | 87 | 0.00728893 | 2.5761E-08 | 2.33032221 | 0.00985292 | 0.06325567 | 282940.545 | 18.1101394 |
| 262 | cycloserine | 258 | 15.2032,0 | 29 | 128 | 0.11748642 | 0.01037505 | 1.43011581 | 0.00280865 | 0.03969122 | 11.3239365 | 3.501303653 |
| 79 | Analyte 88 | 255 | 9.18552,0 | 35 | 221 | 0.00683102 | 0.00454006 | 1.49244979 | 0.02384333 | 0.0979267 | 1.50460931 | 0.589388918 |
| 220 | Analyte 246 | 242 | 13.7768,0 | 36 | 245 | 0.01105002 | 0.02658294 | 2.0935853 | 0.0003608 | 0.01137636 | 0.41568078 | -1.266452037 |
| 81 | Analyte 90 | 237 | 9.24218,0 | 21 | 86 | 0.00270022 | 0.00070095 | 1.31777772 | 0.02157818 | 0.09371603 | 3.85223635 | 1.945696219 |
| 31 | Analyte 34 | 234 | 7.11961,0 | 36 | 207 | 0.02047058 | 0.01673367 | 1.44174523 | 0.01967603 | 0.08978738 | 1.2233172 | 0.290798535 |
| 305 | unknown | 234 | 17.0068,0 | 28 | 160 | 0.04942821 | 0.00458336 | 1.27507378 | 0.04059951 | 0.13822171 | 10.7842775 | 3.430857621 |
| 84 | Analyte 93 | 220 | 9.36304,0 | 28 | 86 | 0.00478766 | 0.00162139 | 1.48954536 | 0.00569534 | 0.05183818 | 2.95281793 | 1.562092405 |
| 387 | unknown | 218 | 19.3473,0 | 15 | 70 | 0.02091725 | 2.5761E-08 | 1.97879197 | 0.00799021 | 0.05755429 | 811962.209 | 19.63105306 |
| 191 | Analyte 214 | 212 | 13.1575,0 | 18 | 128 | 0.53499245 | 2.5761E-08 | 1.96836212 | 0.00545206 | 0.05115844 | 20767244.1 | 24.30780644 |
| 89 | N-cyclohexylformamide 2 | 208 | 9.55167,0 | 36 | 227 | 0.0157729 | 0.00551737 | 2.18192236 | 0.00231775 | 0.03659201 | 2.85876889 | 1.515393995 |
| 503 | saccharopine 2 | 207 | 22.646,0 | 22 | 168 | 2.4558E-08 | 0.18216406 | 1.66687389 | 0.02638488 | 0.10223736 | 1.3481E-07 | -22.82255398 |
| 361 | Analyte 405 | 172 | 18.6211,0 | 14 | 288 | 0.00204652 | 2.5761E-08 | 1.61916763 | 0.0305963 | 0.11389429 | 79441.5558 | 16.27760626 |
| 5 | Analyte 6 | 0 | 6.19019,0 | 36 | 152 | 0.01210291 | 0.01621612 | 1.69749265 | 0.00711042 | 0.05510357 | 0.74635026 | -0.422075256 |
| 6 | Analyte 7 | 0 | 6.21361,0 | 36 | 147 | 0.020408 | 0.02912727 | 1.68712099 | 0.01613954 | 0.08156513 | 0.70064933 | -0.513235533 |
| 17 | Analyte 19 | 0 | 6.66507,0 | 24 | 121 | 0.05814089 | 0.00756819 | 2.03023755 | 0.00028894 | 0.01054289 | 7.68226955 | 2.941532585 |
| 47 | Analyte 51 | 0 | 8.05352,0 | 36 | 73 | 0.13332631 | 0.16737235 | 1.73203911 | 0.00603413 | 0.05271955 | 0.79658503 | -0.328099728 |
| 198 | Analyte 221 | 0 | 13.3183,0 | 35 | 217 | 0.014036 | 0.01984652 | 1.78559685 | 0.00488493 | 0.04939798 | 0.70722709 | -0.499754564 |

Differentially expressed hepatic metabolites between BLA/HFD. The significantly raised metabolites are indicated in blue, the metabolites significantly down-regulated are in red.

**Supplementary Table 3**

**Supplementary Table 3**  **Differentially Expressed Hepatic Metabolites** **between BBA/HFD**

| ID | Peak | Similarity | R.T. | Count | Mass | MEAN HFD | MEAN  BBA | VIP | P-VALUE | Q-VALUE | FOLD CHANGE | LOG_FOLDCHANGE |
| --- | --- | --- | --- | --- | --- | --- | --- | --- | --- | --- | --- | --- |
| 137 | uracil | 909 | 11.1678,0 | 36 | 99 | 0.40337607 | 0.25486889 | 1.93805841 | 0.00090117 | 0.02472979 | 1.582680677 | 0.662370205 |
| 102 | Ethanolamine | 864 | 10.1674,0 | 36 | 174 | 0.44009625 | 0.2531918 | 1.91277456 | 0.00661234 | 0.05903054 | 1.738193147 | 0.797588402 |
| 63 | 3-hydroxybutyric acid | 862 | 8.66729,0 | 36 | 147 | 0.76290899 | 0.48349801 | 1.4645823 | 0.02492681 | 0.1232216 | 1.577894794 | 0.658001017 |
| 189 | L-Malic acid | 862 | 13.096,0 | 36 | 147 | 0.67120652 | 0.53351071 | 1.45431206 | 0.02138467 | 0.11237307 | 1.258093806 | 0.331239496 |
| 28 | 2-hydroxypyridine | 853 | 6.94215,0 | 36 | 152 | 0.70276956 | 0.30315948 | 2.16118894 | 0.00250792 | 0.04085678 | 2.318151378 | 1.212974779 |
| 199 | asparagine 4 | 844 | 13.3522,0 | 36 | 115 | 0.36717214 | 0.27779598 | 1.59309251 | 0.0204307 | 0.10967261 | 1.321733072 | 0.40243085 |
| 205 | methionine 1 | 822 | 13.501,0 | 36 | 176 | 0.23154893 | 0.38657956 | 2.22671188 | 6.4664E-06 | 0.00159704 | 0.598968391 | -0.739448224 |
| 146 | serine 1 | 811 | 11.4611,0 | 26 | 340 | 0.00280166 | 0.00972822 | 1.16582735 | 0.04659855 | 0.17196648 | 0.287993607 | -1.795891308 |
| 212 | 4-aminobutyric acid 1 | 806 | 13.642,0 | 36 | 174 | 0.19280007 | 0.11548124 | 1.62340466 | 0.0112332 | 0.07694954 | 1.669535864 | 0.739447085 |
| 363 | pantothenic acid | 795 | 18.6864,0 | 36 | 201 | 0.02915901 | 0.01888047 | 1.79608467 | 0.00415582 | 0.04778747 | 1.544400227 | 0.627046671 |
| 246 | glutamic acid | 791 | 14.7007,0 | 36 | 246 | 0.5165212 | 0.2835106 | 1.80744379 | 0.00189099 | 0.03650124 | 1.821876185 | 0.865424917 |
| 155 | threonine 1 | 781 | 11.7895,0 | 35 | 73 | 2.36465353 | 1.81648994 | 1.78303596 | 0.0037949 | 0.04664228 | 1.301770787 | 0.380475444 |
| 302 | ornithine 1 | 781 | 16.8938,0 | 36 | 142 | 1.5910542 | 1.34626498 | 1.38445895 | 0.03648461 | 0.15182263 | 1.181828404 | 0.241020579 |
| 86 | valine | 778 | 9.46488,0 | 36 | 144 | 1.8404222 | 1.47630763 | 1.26459507 | 0.04336974 | 0.16604966 | 1.24663868 | 0.318043382 |
| 266 | ribose | 771 | 15.3364,0 | 36 | 307 | 0.41654242 | 0.3226047 | 1.62421092 | 0.01175852 | 0.07848894 | 1.29118522 | 0.36869597 |
| 224 | L-cysteine | 760 | 13.9296,0 | 36 | 220 | 0.08646222 | 0.1591763 | 2.07314948 | 6.5162E-05 | 0.00531065 | 0.54318525 | -0.88048379 |
| 368 | gluconic acid 1 | 743 | 18.8628,0 | 36 | 292 | 0.01966436 | 0.01355026 | 1.66165993 | 0.02128011 | 0.11208267 | 1.451216291 | 0.537262556 |
| 61 | 3-Hydroxypyridine | 711 | 8.54356,0 | 18 | 152 | 0.06331847 | 2.4261E-08 | 1.97155282 | 0.00676008 | 0.05962816 | 2609885.637 | 21.31555516 |
| 364 | unknown | 604 | 18.7368,0 | 34 | 217 | 0.02166302 | 0.0105238 | 1.74664148 | 0.00386794 | 0.04688659 | 2.058478867 | 1.041578637 |
| 309 | unknown | 515 | 17.2394,0 | 20 | 191 | 0.01342986 | 0.0021503 | 1.17653728 | 0.01586348 | 0.09494686 | 6.245579895 | 2.642835531 |
| 143 | unknown | 514 | 11.4377,0 | 36 | 241 | 0.02348732 | 0.05607364 | 2.0967721 | 0.00440369 | 0.04849214 | 0.418865614 | -1.255440641 |
| 179 | 3-Aminoisobutyric acid 1 | 500 | 12.7633,0 | 35 | 174 | 0.03124736 | 0.04329875 | 1.89068682 | 0.00245814 | 0.04055635 | 0.721668969 | -0.470590873 |
| 237 | beta-Glutamic acid 1 | 442 | 14.4764,0 | 36 | 188 | 0.01968919 | 0.0707588 | 2.10615787 | 0.00244181 | 0.04045617 | 0.278257753 | -1.845506207 |
| 295 | methionine sulfoxide 2 | 431 | 16.5453,0 | 34 | 128 | 0.10884519 | 0.02115339 | 2.32714686 | 0.00010473 | 0.00646648 | 5.145519339 | 2.363316696 |
| 243 | unknown | 417 | 14.6178,0 | 18 | 232 | 0.00154847 | 0.011211 | 1.7152078 | 0.00051828 | 0.01776093 | 0.138120578 | -2.85599982 |
| 306 | unknown | 417 | 17.1158,0 | 18 | 204 | 0.02131669 | 2.4261E-08 | 2.08182347 | 0.00583785 | 0.05564497 | 878639.6491 | 19.74491208 |
| 360 | N-alpha-Acetyl-L-ornithine 1 | 402 | 18.613,0 | 36 | 174 | 0.02563419 | 0.04343802 | 2.06611608 | 0.00026053 | 0.01072425 | 0.590132481 | -0.760889227 |
| 206 | unknown | 399 | 13.5236,0 | 27 | 305 | 0.0038124 | 0.01063728 | 1.70149297 | 0.04079072 | 0.16099512 | 0.358399854 | -1.480358044 |
| 62 | Analyte 69 | 392 | 8.63944,0 | 27 | 89 | 0.06886235 | 0.01488692 | 1.18244027 | 0.02835877 | 0.13257253 | 4.625694786 | 2.209670077 |
| 493 | Abietic Acid 2 | 392 | 22.2528,0 | 36 | 79 | 0.01831725 | 0.0270279 | 1.56730186 | 0.02614758 | 0.12666842 | 0.677716284 | -0.561246659 |
| 592 | 5-alpha-Cholestan-3-one 1 | 390 | 28.7333,0 | 23 | 129 | 0.00035331 | 0.00706852 | 1.63794067 | 0.01095435 | 0.07609913 | 0.049983268 | -4.322410947 |
| 83 | malonic acid 1 | 384 | 9.31636,0 | 35 | 147 | 0.01141606 | 0.00719707 | 2.00364317 | 0.00296484 | 0.04329736 | 1.586209023 | 0.665582895 |
| 160 | DL-Anabasine 1 | 382 | 12.0065,0 | 27 | 239 | 0.01061283 | 0.00296192 | 2.11950164 | 0.00875302 | 0.06843647 | 3.583096299 | 1.841206818 |
| 568 | Lignoceric acid | 370 | 25.7586,0 | 24 | 103 | 0.00590768 | 0.01235955 | 1.0120489 | 0.04530605 | 0.16965002 | 0.477984978 | -1.064962816 |
| 151 | 3-Cyanoalanine | 365 | 11.6733,0 | 34 | 141 | 0.01614418 | 0.01223075 | 1.46326474 | 0.0316413 | 0.1406128 | 1.319966143 | 0.400500925 |
| 67 | unknown | 361 | 8.75359,0 | 36 | 281 | 0.01873529 | 0.02219757 | 1.42150679 | 0.04436341 | 0.16791726 | 0.844024334 | -0.244643502 |
| 49 | Analyte 53 | 332 | 8.14454,0 | 36 | 245 | 0.02132492 | 0.01261276 | 1.66793803 | 0.02027932 | 0.10923336 | 1.690741488 | 0.757656091 |
| 66 | unknown | 325 | 8.74023,0 | 36 | 128 | 0.22236875 | 0.05122794 | 2.24406695 | 0.00175022 | 0.03527176 | 4.340771106 | 2.11795135 |
| 419 | unknown | 313 | 20.219,0 | 24 | 299 | 2.4558E-08 | 0.0050726 | 2.02664454 | 0.00993847 | 0.07278675 | 4.84126E-06 | -17.65618497 |
| 166 | Biuret 3 | 311 | 12.2171,0 | 30 | 86 | 0.015528 | 0.00378617 | 1.16575 | 0.00016703 | 0.00825034 | 4.101246626 | 2.036062502 |
| 474 | glutathione 2 | 307 | 21.6393,0 | 31 | 101 | 0.02030028 | 0.04542162 | 1.72109061 | 0.00244058 | 0.04044858 | 0.446929881 | -1.161879591 |
| 103 | Analyte 113 | 304 | 10.1851,0 | 33 | 319 | 0.02185123 | 0.01702837 | 1.54458173 | 0.01931913 | 0.1063755 | 1.283224729 | 0.359773849 |
| 133 | oxamide | 299 | 11.0945,0 | 33 | 215 | 0.02894539 | 0.00927559 | 2.22646742 | 2.311E-05 | 0.00285381 | 3.120599497 | 1.641823211 |
| 227 | unknown | 295 | 14.0406,0 | 23 | 244 | 0.00426798 | 0.00153593 | 1.48500465 | 0.01124334 | 0.07698001 | 2.778767194 | 1.47444497 |
| 369 | unknown | 271 | 18.8693,0 | 31 | 283 | 0.00666372 | 0.00344301 | 1.06442092 | 0.04776683 | 0.17400339 | 1.935433341 | 0.95265662 |
| 180 | Citraconic acid 3 | 258 | 12.7741,0 | 18 | 87 | 0.00728893 | 0.00079673 | 2.043658 | 0.00805927 | 0.06560667 | 9.14853125 | 3.193540145 |
| 262 | cycloserine | 258 | 15.2032,0 | 29 | 128 | 0.11748642 | 0.01833419 | 2.26857522 | 0.00423456 | 0.048018 | 6.408049502 | 2.679885293 |
| 79 | Analyte 88 | 255 | 9.18552,0 | 35 | 221 | 0.00683102 | 0.00481207 | 1.46456663 | 0.04330165 | 0.16592009 | 1.419558866 | 0.505442676 |
| 220 | Analyte 246 | 242 | 13.7768,0 | 36 | 245 | 0.01105002 | 0.0179844 | 1.52139029 | 0.03299248 | 0.14369627 | 0.614422424 | -0.702697225 |
| 73 | Analyte 82 | 241 | 8.94562,0 | 35 | 221 | 0.00766491 | 0.00571798 | 1.46163364 | 0.02986047 | 0.13635208 | 1.340491636 | 0.422762218 |
| 31 | Analyte 34 | 234 | 7.11961,0 | 36 | 207 | 0.02047058 | 0.01597191 | 1.64481338 | 0.0085721 | 0.06772032 | 1.2816612 | 0.358014944 |
| 387 | unknown | 218 | 19.3473,0 | 15 | 70 | 0.02091725 | 0.00172758 | 1.66475705 | 0.00957993 | 0.07153024 | 12.10780571 | 3.597865525 |
| 584 | Analyte 659 | 216 | 27.7991,0 | 21 | 73 | 0.00193898 | 0.01373844 | 1.25797759 | 0.00672361 | 0.05948197 | 0.141135331 | -2.824848907 |
| 191 | Analyte 214 | 212 | 13.1575,0 | 18 | 128 | 0.53499245 | 2.4261E-08 | 1.97191883 | 0.00545206 | 0.05378154 | 22051530.01 | 24.39437542 |
| 89 | N-cyclohexylformamide 2 | 208 | 9.55167,0 | 36 | 227 | 0.0157729 | 0.00676694 | 2.12475303 | 0.00357964 | 0.04588093 | 2.330876794 | 1.220872748 |
| 503 | saccharopine 2 | 207 | 22.646,0 | 22 | 168 | 2.4558E-08 | 0.20787146 | 2.3739776 | 0.00085662 | 0.02406518 | 1.18139E-07 | -23.01300729 |
| 361 | Analyte 405 | 172 | 18.6211,0 | 14 | 288 | 0.00204652 | 2.4261E-08 | 1.6887777 | 0.03059622 | 0.13814052 | 84354.37297 | 16.36417524 |
| 5 | Analyte 6 | 0 | 6.19019,0 | 36 | 152 | 0.01210291 | 0.01640805 | 1.63959303 | 0.01426754 | 0.08896939 | 0.737620212 | -0.439049906 |
| 17 | Analyte 19 | 0 | 6.66507,0 | 24 | 121 | 0.05814089 | 0.01973759 | 1.4364874 | 0.00455271 | 0.04888775 | 2.945694087 | 1.558607613 |
| 47 | Analyte 51 | 0 | 8.05352,0 | 36 | 73 | 0.13332631 | 0.16200278 | 1.60156076 | 0.01954224 | 0.10705082 | 0.82298779 | -0.281057068 |
| 198 | Analyte 221 | 0 | 13.3183,0 | 35 | 217 | 0.014036 | 0.01785716 | 1.50665219 | 0.04803464 | 0.17446295 | 0.786014801 | -0.347371615 |

Differentially expressed hepatic metabolites between BBA/HFD. The significantly raised metabolites are indicated in blue, the metabolites significantly down-regulated are in red.
